# Detection of active and inactive phases of thyroid-associated ophthalmopathy using deep convolutional neural network

**DOI:** 10.1186/s12886-020-01783-5

**Published:** 2021-01-14

**Authors:** Chenyi Lin, Xuefei Song, Lunhao Li, Yinwei Li, Mengda Jiang, Rou Sun, Huifang Zhou, Xianqun Fan

**Affiliations:** 1 Department of Ophthalmology, Shanghai Ninth People’s Hospital, Shanghai Jiao Tong University School of Medicine, 639 Zhi Zao Ju Road, Shanghai, 200011 China; 2 Shanghai Key Laboratory of Orbital Diseases and Ocular Oncology, Shanghai, China

**Keywords:** Machine learning, Thyroid-associated ophthalmopathy, Magnetic resonance imaging

## Abstract

**Background:**

This study aimed to establish a deep learning system for detecting the active and inactive phases of thyroid-associated ophthalmopathy (TAO) using magnetic resonance imaging (MRI). This system could provide faster, more accurate, and more objective assessments across populations.

**Methods:**

A total of 160 MRI images of patients with TAO, who visited the Ophthalmology Clinic of the Ninth People’s Hospital, were retrospectively obtained for this study. Of these, 80% were used for training and validation, and 20% were used for testing. The deep learning system, based on deep convolutional neural network, was established to distinguish patients with active phase from those with inactive phase. The accuracy, precision, sensitivity, specificity, F1 score and area under the receiver operating characteristic curve were analyzed. Besides, visualization method was applied to explain the operation of the networks.

**Results:**

Network A inherited from Visual Geometry Group network. The accuracy, specificity and sensitivity were 0.863±0.055, 0.896±0.042 and 0.750±0.136 respectively. Due to the recurring phenomenon of vanishing gradient during the training process of network A, we added parts of Residual Neural Network to build network B. After modification, network B improved the sensitivity (0.821±0.021) while maintaining a good accuracy (0.855±0.018) and a good specificity (0.865±0.021).

**Conclusions:**

The deep convolutional neural network could automatically detect the activity of TAO from MRI images with strong robustness, less subjective judgment, and less measurement error. This system could standardize the diagnostic process and speed up the treatment decision making for TAO.

## Synopsis

The study proposed a method based on deep convolutional neural network to detect the activity of thyroid-associated ophthalmopathy from orbital magnetic resonance imaging, with high accuracy, sensitivity and specificity.

## Background

TAO, an autoimmune disease associated with thyroid disease, is the most common orbital disease in adults. TAO not only affects the appearance of patients but may also impair visual function seriously [1]. Two phases in the development of TAO are generally distinguished: the active phase and the inactive phase [2]. In the active phase, patients feel red and painful in eyes because of vasodilatation and inflammatory cell infiltration caused by active inflammation, and respond well to immunosuppressive therapy. In the inactive phase, with fibrosis taking place, patients show painless motility deficit in eyes, and immunosuppressive therapy is useless [3]. Clinical activity score (CAS) is a common index of activity staging based on typical inflammatory manifestations [2]. Without using special instruments, CAS is easily manageable and increases the success rate of immunosuppressive therapy. However, because it is entirely clinical, this index is less sensitive to disease progression in subclinical patients and during treatment [4, 5]. Besides, Wang proposed that because of the differences in orbital anatomy between Caucasians and Asians, cut-off point for the CAS of Asian patients might be lower than the standard [6].

MRI is another way to evaluate the activity of TAO. Orbital MRI can clearly reflect the structure and pathology of the orbit, with no ionizing radiation, high contrast of soft tissue, and multi-parameter imaging. The intraorbital signal intensity of patients with active TAO was significantly different from that of inactive patients in several MRI sequences, such as short TI inversion recovery sequence (STIR), diffusion tensor imaging, and dynamic enhancement techniques [5–8]. Politi found that orbital MRI might be more sensitive than clinical examinations and could become biomarkers of early asymptomatic inflammatory [9]. However, the disease-related information contained in orbital MRI is not fully deciphered, and film reading is mostly manual reading in clinical work, which relies on the accumulation of experience. Therefore, the complex multi-dimensional information of orbital MRI can only be transformed into simple and measurable information before it can be understood and used, such as diameter, signal intensity and so on. Furthermore, the slight change of signal intensity caused by mild inflammation is easily overlooked by ophthalmologists who do not specialize in TAO. This feature engineering could lead to the loss of effective information and even misdiagnosis.

In view of the shortcomings above, it is necessary to develop a sensitive and accurate method for activity staging of Asian patients, which is not limited to professional experience. Deep learning, an end-to-end machine learning method, is not limited by prior knowledge [10]. As one of deep learning, deep convolutional neural network (DCNN), which could extract features automatically and classify accurately, has been widely used in medical fields recently, including ophthalmology. There were researches of DCNNs based on digital retinal photos and optical coherence tomography conducted to identify anterior segment disease and fundus disease [10–20]. However, studies on the recognition of orbital diseases based on orbital MRI have not been reported.

In this study, a deep learning system based on DCNN was established to recognize orbital MRI for differentiating the active phase from the inactive phase in patients with TAO. In the rest of the paper we introduced the subject and the database, described the structure of the network we built and evaluated the performance through accuracy, precision, sensitivity, specificity, F1 score and area under the receiver operating characteristic curve.

## Methods

### Patients

This study followed the tenets of the declaration of Helsinki and was approved by the ethics committee of Shanghai Ninth Peoples’ Hospital (Approval No. SH9H-2018-T41–2). Patients who came to the Ophthalmology Clinic of the Ninth People’s Hospital from May 01, 2018, to July 01, 2019, were retrospectively examined. The inclusion criteria were as follows: (1) age more than 18 years, (2) meeting the internationally recognized diagnostic criteria for TAO, and (3) intraorbital involvement confirmed by orbital MRI. European Group on Graves’ orbitopathy (EUGOGO) suggested that the severity of TAO could be divided into mild, moderate, and extremely severe according to the degree of eyelid retraction, exophthalmos, diplopia, and other indices [1]. The eyes with higher severity were analyzed in the included patients. The exclusion criteria were as follows: (1) eyelid retraction, exophthalmos, and eye movement disorders caused by other eye diseases and (2) patients with metal implants or mental illness. The data types included basic information of patients, physical examination, auxiliary examination and treatment suggestion. Meanwhile, the therapeutic effect and adverse events were recorded during treatment.

A total of 108 patients were included in this study. Among patients, there were 66 females and 42 males. Patients in active phase were treated with immunosuppressive therapy. One hundred sixty orbital MRIs were performed before and after treatment. Due to the lack of data and the significant change of MRI signal intensity before and after treatment, we regarded the data before and after treatment as independent data.

### Data preparation

#### Acquisition of orbital MRI

MRI scanning was performed on a 3.0-T MRI system (Philips Ingenia 3.0 T). Throughout the scan, the patient’s head was stabilized in a supine position and kept stationary. The orbital MRI imaging protocol included T1-weighted turbo spin-echo (TSE), T2-weighted TSE spectral presaturation with inversion recovery (SPIR), and T2-weighted driven equilibrium radiofrequency reset pulse (DRIVE). Axial SPIR was chosen for the present study (repetition time/echo time = 3000.0/80.0; number of sample acquisition = 1.6; field of view = 120 × 150 × 49 mm3; slice thickness = 3 mm; matrix = 240 × 226 × 15).

#### Data annotation

The DCNN used in this study belongs to the supervised learning method. CAS is the most commonly used clinical staging index of TAO, whose assessment includes 7 clinical manifestations: spontaneous retrobulbar pain, pain on attempted upward or downward gaze, redness of eyelids, redness of conjunctiva, swelling of caruncle or plica, swelling of eyelids and swelling of eyelids. We used CAS to annotate the activity stage of orbital MRI [5]. Patients with CAS ≥3/7 were annotated as active phase, while patients with CAS< 3/7 were annotated as inactive phase. In this study, the activity of TAO was assessed based on CAS score by two ophthalmologists with more than 5-year experience in orbital diseases (Xuefei Song and Lunhao Li). Disagreement, if any, was resolved by a senior chief physician (Huifang Zhou) after an empirical evaluation of orbital MRI. After annotation, there were 50 MRI images of active phase and 110 MRI of inactive phase.

#### Dataset division

The dataset for testing was generated by a random split of 20% of the entire set, which didn’t participate in training process. A 32-image dataset was generated for testing, among which there were 7 MRI images of active phase and 25 MRI of inactive phase. K-fold cross-validation was employed to the remaining dataset (training set) for hyper-parameter tuning (k = 5). This algorithm divided the data into k folds randomly without replacement. During one cross-validation process, k-1 folds were used for training, and the remaining fold was used for validation. This process was then repeated k times to use each of the k folds once for validation. After finding the satisfactory hyper-parameters through K-fold cross-validation, A new model was trained from the whole training set.

#### Data normalization and augmentation

Intensity nonuniformity in MRI, also known as bias field, was caused by the location of patients, scanner parameters, and environment. This difference in signal intensity has nothing to do with anatomical and physiological factor, which may reduce the comparability of MRI images. N4 bias field correction was carried out to reduce this inconsistency [21]. After correction, the pixels of each image were normalized to an average of 0 and a standard deviation of 1. Finally, the orbital parts of MRI were intercepted with ITK-SNAP 3.6.0 and zoomed to 164 × 164 pixel size [22]. With the optic nerve plane as the center, five slices of MRI images were chosen.

**Fig. 1:**
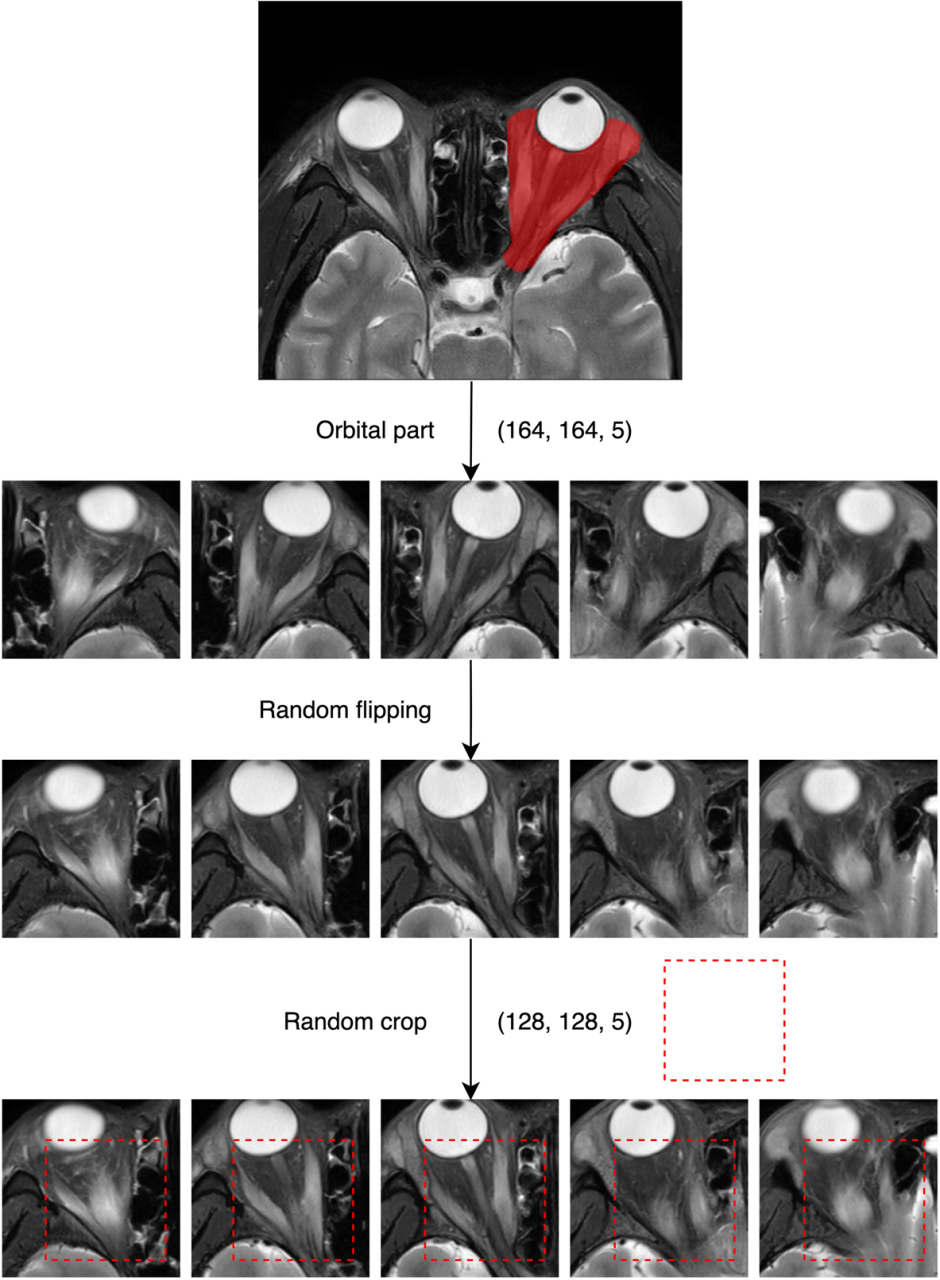
Data preprocessing and data augmentation. Orbital part of the MRI image was captured using ITK-SNAP after normalization and N4 bias field correction (164, 164, 5). Captured images were augmented by random flipping and crop and entered the neural network (128, 128, 5)

To avoid overfitting, data enhancement techniques were used before each training, including random flipping and random crop, after which the size of the MRI became 128 × 128 × 5 pixels. Figure 1 shows the process of data preprocessing and data augmentation.

### Deep convolutional neural network

**Fig. 2:**
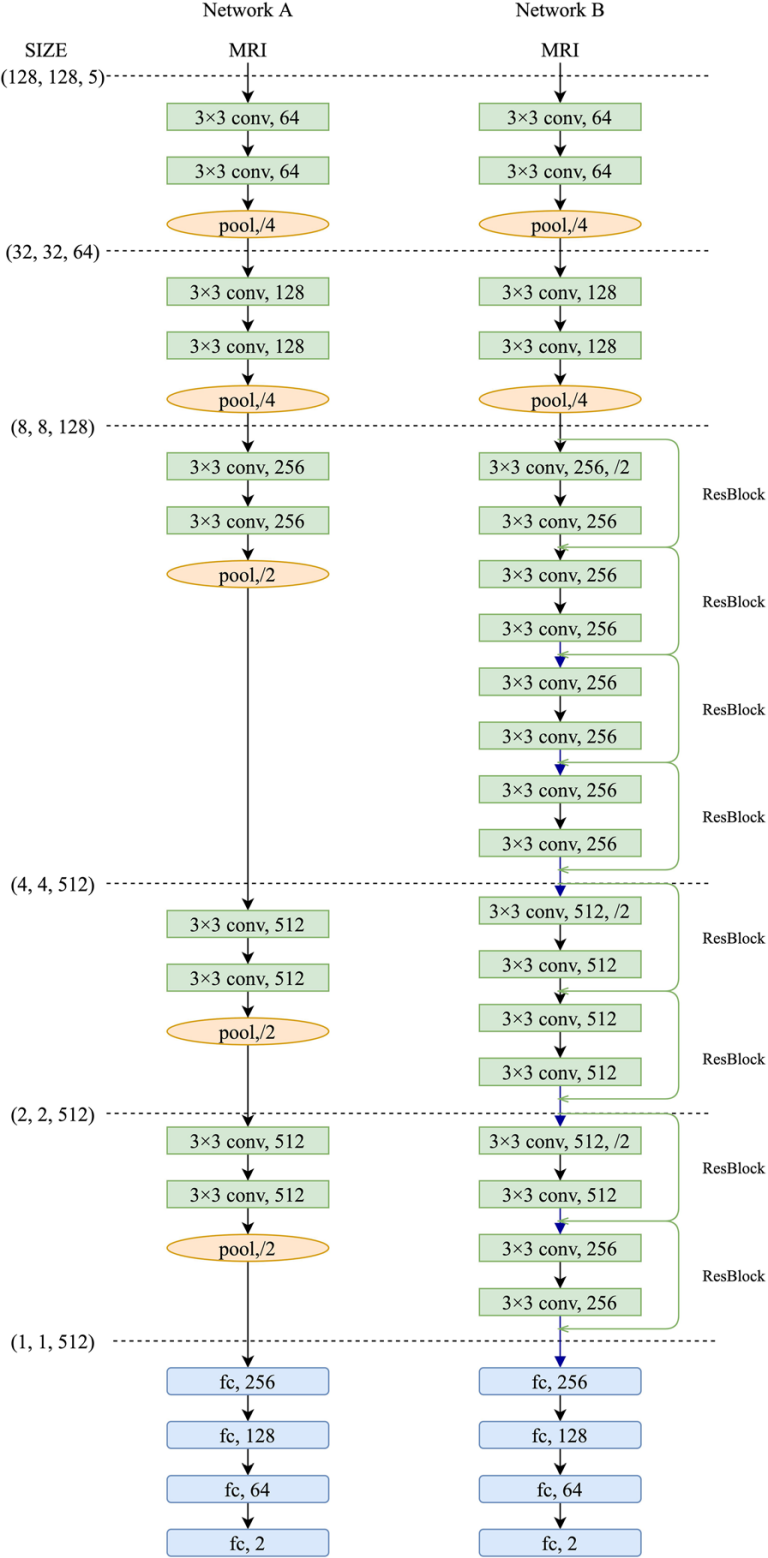
Sketch maps of the networks. Network A, inherited from VGG-16, consisted of five convolution modules and four full connection layers. Each module contained two convolution layers (conv) and one max pooling layer (pool). On the basis of network A, network B added eight ResBlock modules with two convolution and one residual path (green line)

The basic DCNN consisted of convolution layer, pooling layer and fully connected layer. Convolution layer could extract features by filters. In order to avoid the overfitting by the increase of features after several filters, pooling layer was proposed to subsample and to reduce the number of parameters. Besides, this layer could retain the relative invariance of space. After obtaining all the features, the fully connection layer was used to integrate them to complete the classification task. In addition to the basic structure of DCNN, we added nonlinear activation functions, batch normalization, dropout layer and softmax function to optimize the network. Rectified linear unit (ReLU) was applied as nonlinear activation function after every convolution layer and every fully connected layer, which could speed up the convergence by sparse activation. Between the convolution layer and the activation function, batch normalization was set up to normalize the distribution of each batch, which could effectively reduce the phenomenon of gradient disappearance [23]. Dropout layer dropped a part of parameters randomly with probability 1-p, which could prevent overfitting by simplify the network (*p*=0.5). Softmax function, following fully connected layer, could convert the output to a probability distribution. Our proposed DCNNs inherited parts of Visual Geometry Group (VGG) network and Residual Neural Network (ResNet), structures of which are shown in Fig. 2. Network A inherited VGG network, with small filters (3× 3) to reduce the complexity. Network A had five convolutional modules and four full connection layers. Each convolutional module contained two convolutional layers and one max pooling layer. On the basis of network A, network B inherited ResNet, with eight ResBlock modules to increase the depth and to reduce the occurrence of vanishing gradient and exploding gradient. Each ResBlock contained two convolutional layers and one residual path. During the training process, Adam optimizer, learning rate decay, weight decay and momentum optimization were applied to accelerate the convergence and to improve training speed [24]. Cross-entropy was selected as the loss function. Detailed adjustments of hyperparameters are shown in Table 1. The entire process was carried out on the machine MECHREVO MR LX980 equipped with RTX2080, using the Google’s TensorFlow2.0 as the backend.

**Table 1:** Detailed adjustments of hyperparameters

Hyperparameters	Details	
	Network A	Network B
Depth	10 convolution layers	20 convolution layers
Activation function	Rectified linear unit (ReLU)	
Loss function	Cross-entropy	
Filter size	3×3, channels increased from 64 to 128 to 256 to 512, stride was 1 or 2	
Batch size	64	
Epoch	300	400
Learning rate	3×10^−4^, decay to 1×10^−4^ after 250 epoches	3× 10^− 4^, decay to 5× 10^−5^ after 300 epoches
Optimizer	Adam optimizer (β_1_=0.9, β_2_=0.999) with momentum	
Batch normalization	After every convolution layer	
Regularization	Weight decay by L2 regularization (λ=0.01 in convolution layer, λ=0.001 in fully connected layer)	

### Evaluation

The experiment was run four times to evaluate the stability of training. The convergence of loss value in training was achieved to evaluate the training process. The preprocessed image was input into the well-trained networks, the probability was calculated, and a decision was output. The average and standard deviation (SD) of accuracy, precision, sensitivity, specificity and F1 score of four runs on test set were calculated, the definitions of which were given by:

$$ Accuracy=\frac{TP+ TN}{TP+ TN+ FP+ FN} $$

$$ Precision=\frac{TP}{TP+ FP} $$

$$ Sensitivity=\frac{TP}{TP+ FN} $$

$$ Specificity=\frac{TN}{TN+ FP} $$

$$ F1\  score=\frac{2\times Precision\times Sensitivity}{Precision+ Sensitivity} $$

F1 score is an index to measure the accuracy of binary classification model, which conveys the balance between precision and sensitivity. The effectiveness was also evaluated by drawing the receiver operating characteristic (ROC) curve and calculating the area under curve (AUC).

In addition, it is difficult to tell how neural network works, so the occlusion testing, a kind of visualization method, was applied to reveal insights into the decisions of neural networks. We set gray occlusion region on the input image, and recorded the output probability while different regions were occluded. By converting these probability changes into heat map, the key parts of the input image for decision could be displayed.

## Results

**Fig. 3:**
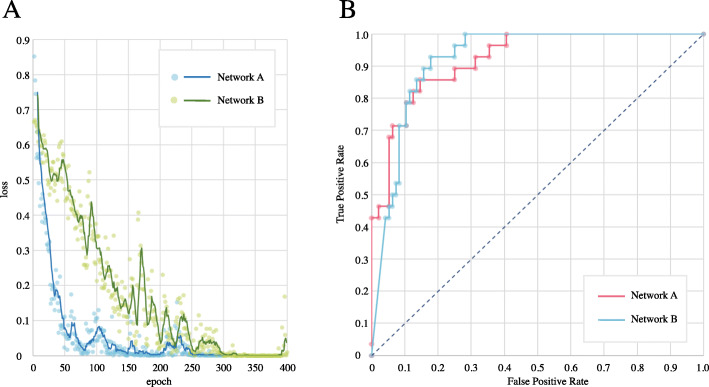
**a**. The convergence of loss value of network A (blue) and network B (green). The point represented the original data, and the line represented the fitting curve. **b**. Receiver operating characteristic curve of network A (red) and network B (blue). AUC of both networks was 0.922

**Fig. 4:**
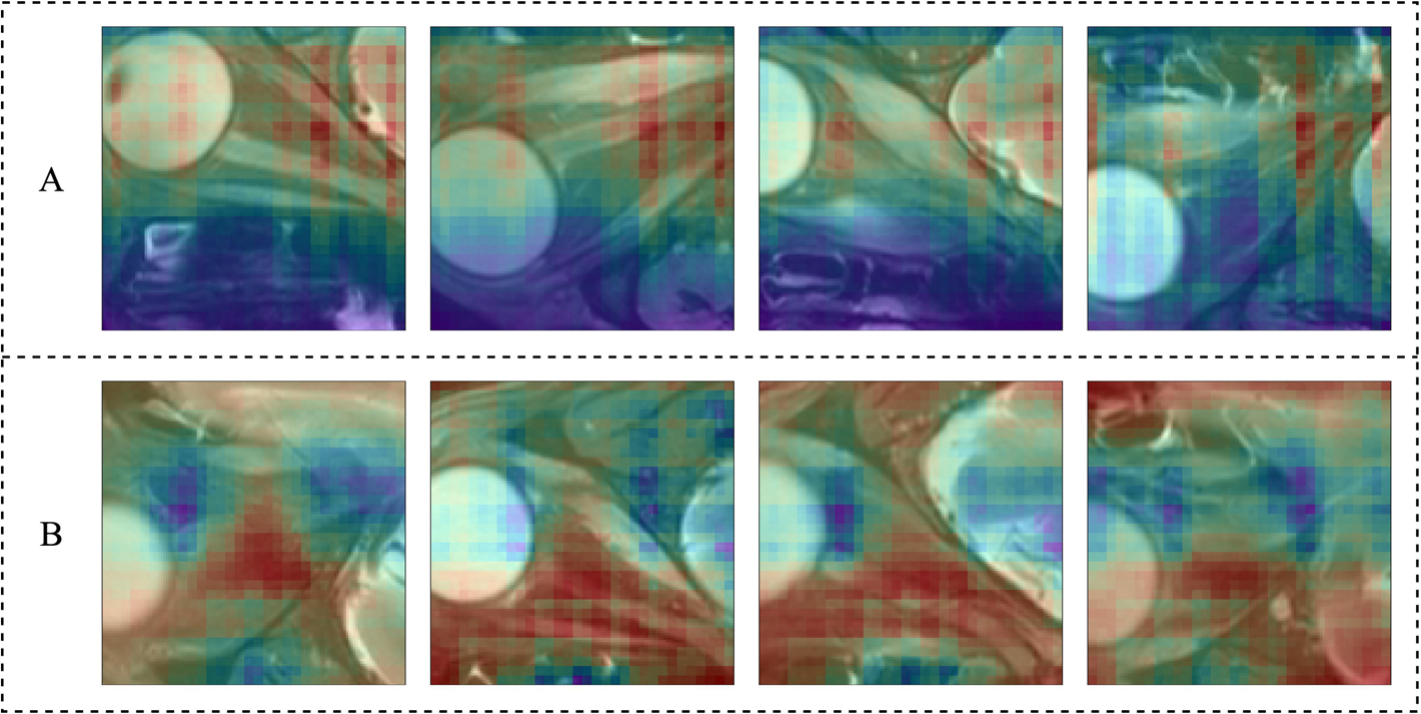
Heatmap of occlusion testing of network A (up) and network B (down). High heat colors, such as red and orange, were the key parts in orbital MRI for clinical staging of TAO

The experiment was run four times on both networks. The results of accuracy, precision, sensitivity, specificity and F1 score of each network were summarized in Table 2, shown as average±SD. Both networks got high accuracy (network A: 0.863±0.055, network B: 0.855±0.018) and performed well on other indicators. The SD of five measures in network B was smaller than that in network A, which indicated that the training of network B was more stable. Figure 3 shows the convergence of loss value during training process and the ROC during testing process. The loss values of both networks could nearly converge to 0, in which network A did faster. The AUC value of both networks was 0.922. Figure 4 shows the results of occlusion testing by heat map. Each region was marked with thermal color according to the probability change caused by its occlusion. High-heat colors in the images were the key parts for decision, such as red and yellow. They mainly located on the edematous extraocular muscles.

**Table 2:** Evaluation of the networks

	Accuracy	Precision	Sensitivity	Specificity	F1 score
Network A	0.863 ± 0.055	0.680 ± 0.124	0.750 ± 0.136	0.896 ± 0.042	0.712 ± 0.121
Network B	0.855 ± 0.018	0.640 ± 0.033	0.821 ± 0.071	0.865 ± 0.021	0.719 ± 0.040

## Discussion

TAO is the most common orbital disease in adults. Its clinical staging is the main index to guide treatment. The existing CAS is entirely clinical, which makes it less sensitive to disease progression in subclinical patients and during treatment [5]. Orbital MRI has been used to assist in clinical staging. The challenges in an MRI-based clinical application include the selection of sequences, signal inconsistencies and data protection. T2-weighted sequence of MRI can reflect the water content of tissue, so as to distinguish inflammatory edema from fibrous hyperplasia. In order to reduce the interference of fat signal, T2-weighted sequence is often combined with fat suppression technique, such as STIR and SPIR. Higashiyama pointed out that the signal intensity rate of extraocular muscle in STIR sequence was positively correlated with CAS score and was a positive predictor of treatment response, related to the risk of deterioration [3, 25]. In this study, SPIR sequence was selected for training and testing. To reduce signal inconsistencies, N4 bias field correction was applied. However, MRI film reading depends on experience and may loss effective information. Deep learning is not limited by experience and DCNN could extract features automatically and classify accurately, which could standardize the process of MRI fim reading.

At present, DCNN based on MRI is restricted to the brain, kidney, prostate and spine. The major technical problems include difficulty in three-dimensional reconstruction, overfitting caused by small datasets, and the “black box” problem [26]. In order to solve the difficulty in three-dimensional reconstruction of MRI images, we treated three-dimensional images as stacks of two-dimensional images in our study. In the face of overfitting caused by small datasets, one of the current research hotspots is transfer learning. Transfer learning is a method transferring the knowledge from some previous tasks to a present target, and is classified to three types: unsupervised transfer learning, inductive transfer learning, and transductive transfer learning [27]. Zhang and Kermany found that transfer learning could speed up training and reduce redundant processes, with good performance in small datasets [28, 29]. However, when the selected source domain was not related to the target domain, negative transfer may happen, which make it perform even worse [27]. As orbital MRI images were similar to each other but not similar to common datasets, we chose to inherit parts of VGG and ResNet rather than transfer learning. Network A inherited VGG network with small filters repeatedly. In this way, the network structure could be simplified, and the task of binary classification could be completed with good generalization ability. Due to the recurring phenomenon of vanishing gradient in the process of training, we added the ResBlock module of ResNet to build network B. After transmitting the original input directly to the following layer by residual path, network B greatly increased the depth, and reduced the occurrence of vanishing gradient and exploding gradient. In addition, weight decay, learning rate decay, momentum and other methods were applied to optimize both networks. To explain how the networks worked, occlusion testing, one of the most commonly used visualization methods, was also applied in this study.

The results shown in Table 2 and Fig. 3 indicated that both networks performed well in four runs. Network A had better accuracy (0.863) and specificity (0.896), while network B had good sensitivity (0.821) and was more stable when training. Both networks had high AUC (0.922). For such a small dataset, the good performance suggested that: (1) SPIR sequence of orbital MRI did contain depth characteristics that reflected the progress of TAO; (2) this binary classification task was in good agreement with the system established. The results of occlusion testing shown in Fig. 4 indicated that the key parts for decision-making mainly located on the edematous extraocular muscle, which was consistent with our cognition.

This study was novel in proposing a DCNN-based deep learning system to stage the activity of TAO. This deep learning system could avoid subjective error and explore in-depth information on intraorbital changes, which could assist ophthalmologists, who do not specialize in TAO, to evaluate the activity. Furthermore, the application of visualization methods may not only assist the staging of the disease, but also have educational significance for clinical beginners, such as indicating swelling and hyperemia of extraocular muscles and so on. However, this study had several limitations: (1) Manual interception of the orbital part was costly. This step will be further automated in the future. (2) Although regularization methods were applied, it was still difficult to completely avoid overfitting for such a small dataset. In further studies, the database should be expanded to increase the robustness of the system. (3) We only chose one MRI sequence for decision making, which may cause bias. Other MRI sequences should be added to look for new indicators and to further explain the role of MRI in clinical staging of TAO. Also, medical imaging data will be combined with treatment and prognosis to establish a sounder intelligent decision-making system. After finding a solution of data access and data protection, telemedicine platforms will be used to promote a new Predictive, Preventive, Personalized, and Participatory (4P) medical paradigm for the diagnosis and treatment of TAO.

## Conclusions

The orbital MRI contains the depth characteristics of TAO clinical staging. DCNN doesn’t rely on subjective judgment and could directly get these features from the orbital MRI to assist the clinical staging of TAO patients, with small measurement error and strong robustness. The networks established in this study performed well during evaluation process. In further studies, we will develop its potential for efficacy evaluation and prediction. Through telemedicine platforms, we could standardize the diagnosis and treatment of TAO and speed up the decision-making process in the future.

## Data Availability

The datasets analyzed during the current study are available from the corresponding author (lynnstaria@sjtu.edu.cn) on reasonable request.

## Abbreviations

TAO: Thyroid-associated ophthalmopathy
MRI: Magnetic resonance imaging
CAS: Clinical activity score
STIR: Short TI inversion recovery sequence
EUGOGO: European Group on Graves’ orbitopathy
TSE: T1-weighted turbo spin-echo
SPIR: Spectral presaturation with inversion recovery
DRIVE: Driven equilibrium radiofrequency reset pulse
DCNN: Deep convolutional neural network
VGG: Visual Geometry Group
ResNet: Residual Neural Network
ROC: Receiver operating characteristic
AUC: Area under curve
TP: True positive
TN: True negative
FP: False positive
FN: False negative
SD: Standard deviation
